# Unraveling gene interaction networks in colorectal cancer and inflammatory bowel disease via a novel hybrid radial basis function network

**DOI:** 10.1038/s41598-025-33847-1

**Published:** 2026-01-23

**Authors:** Duygu Kırkık, Faruk Bulut

**Affiliations:** 1 Hamidiye Medicine Faculty, Department of Immunology, University of Health Sciences, Istanbul, Turkey; 2 School of Computer Science and Electronic Engineering, University of Essex, Colchester, UK; 3 Software Engineering, Istanbul Aydin University, Istanbul, Turkey; 4 Hamidiye Medicine Faculty, Department of Medical Biology, University of Health Sciences, Istanbul, Turkey

**Keywords:** Machine learning, Clustering, Genomics, Immunoinformatics, Colorectal cancer, Biomarkers, Cancer, Computational biology and bioinformatics, Diseases, Gastroenterology, Genetics

## Abstract

Colorectal cancer (CRC) is a significant global health challenge, closely linked with inflammatory bowel disease (IBD). Understanding the genetic and molecular underpinnings of CRC and its association with IBD is critical for early diagnosis and personalized treatment. This study introduces a novel Hybrid Radial Basis Function (RBF) Network approach for gene clustering to uncover key genetic interactions and pathways associated with these conditions. Gene datasets related to CRC and IBD were retrieved from public databases, including OMIM and Entrez Gene. Functional and structural analyses of these genes were conducted using bioinformatics tools such as STRING and GeneMania. A Hybrid RBF Network clustering methodology was employed to analyze gene sequence similarities, leveraging density thresholds, Gaussian functions, and clustering resolution parameters for optimal performance. The clustering quality was evaluated using metrics like the Silhouette Score, Calinski–Harabasz Index, and Davies–Bouldin Index. The study identified central genes such as APC, SMAD4, and MSH2 as critical nodes in the gene interaction network, emphasizing their role in CRC and IBD pathogenesis. The clustering methodology demonstrated superior performance (Silhouette Score: 0.70; Calinski–Harabasz Index: 30.5; Davies-Bouldin Index: 0.50) compared to conventional techniques. Furthermore, interactions between NLRP3 and PYCARD highlighted the potential involvement of inflammasomes in linking chronic inflammation to carcinogenesis. The proposed Hybrid RBF Network approach provides a robust framework for gene clustering and provides new insights into the genetic basis of CRC and IBD. Our work highlights the transformative potential of machine learning and bioinformatics in advancing genomic research and precision medicine.

## Introduction

Colorectal cancer (CRC) is responsible for 10% of new cancer cases globally and is the third most common cancer after prostate and lung cancer in men and breast and thyroid cancer in women^1,2^. The incidence of CRC varies between different geographical regions, and this variation is associated with dietary habits that vary depending on cultural differences^3^. For instance, diets high in processed meats and low in fiber have been consistently linked to higher CRC risk, highlighting the impact of lifestyle factors on disease prevalence.

Recent studies have shown that the risk of CRC is increased in people with IBD^4^. The risk of CRC is closely related to the duration and anatomical location of the IBD. It has been reported that CRC that develop in individuals with IBD have higher mortality rates^5^. This association underscores the need for proactive management strategies, particularly in high-risk groups. Furthermore, the interplay between chronic inflammation and genetic susceptibility in IBD patients offers a unique insight into tumorigenesis. Although there are no comprehensive controlled studies, follow-up in IBD is recommended by the American College of Gastroenterology (ACG)^6^, the American Society for Gastrointestinal Endoscopy (ASGE)^7^, the American Gastroenterological Association (AGA)^8^ and the British Gastroenterological Association^9^. These guidelines emphasize early and regular screening, which has been shown to mitigate cancer risk by enabling timely detection of precancerous lesions.

The primary goal of follow-up in IBDs is the detection of dysplasia, which is strongly associated with CRC^10^. Screening is especially important for patients diagnosed with IBD at a young age. Screening has the potential to reduce cancer risk by detecting and removing precancerous lesions (dysplasia) in the early stages of the disease. Furthermore, screening in IBD is important for early detection and removal of dysplasia, monitoring cancer development and cancer treatment^11^. This proactive approach not only improves patient outcomes but also reduces healthcare burdens associated with advanced cancer management. Recent advancements in bioinformatics have shown the study aimed at understanding the intricate relationship between IBD and CRC^12,13^. Emerging machine learning models and computational tools have revolutionized how we identify genetic markers and pathways associated with disease progression. These technological advances pave the way for innovative diagnostic and therapeutic interventions^14^.

This study primarily aims to use machine learning and bioinformatics to investigate the genetic basis of CRC in patients with IBD. A secondary objective is to identify key biomarkers and develop predictive models to improve early diagnosis and personalize treatment strategies.

## Materials and methods

### Dataset

The National Center for Biotechnology Information (NCBI)11 maintains and hosts the Online Mendelian Inheritance in Man (OMIM) and Entrez Gene databases, two reputable public repositories from which this study is based. The initial data retrieval was guided by specific search criteria that focused on genes with documented, high-confidence associations with Colorectal Cancer (CRC) (including Colitis-Associated Cancer, CAC) and Inflammatory Bowel Disease (IBD) (including Crohn’s Disease and Ulcerative Colitis) in order to ensure biological relevance and reduce selection bias. In the context of pathways linked to chronic inflammation and subsequent oncogenesis, our rigorous methodological approach produced a focused, non-redundant set of 237 distinct genes, offering a scientifically informed and manageable input size for complicated clustering analysis^15,16^.

To convert the raw biological data into a quantitative feature space appropriate for the Radial Basis Function (RBF) Network^17^, a thorough preprocessing pipeline was necessary after capture. The **complete nucleotide sequence** that corresponds to the longest verified transcript for each gene was taken straight from the NCBI data and used as the primary characteristic for clustering. Duplicate entries were eliminated, and sequences that were either incomplete or shorter than a critical length criteria (such as 1000 base pairs) were strictly excluded as part of data integrity tests. This quality control step keeps the downstream k-mer profiles from being distorted by possible data artifacts or noise from sequences with poor information content^18^.

The critical step in numerical encoding was to use **k-mer profiling** to convert the variable-length sequences into a fixed-size representation. For this, a biologically optimal *k* value (e.g., $k=5$) was chosen to maximize the balance between sequence specificity and feature dimension manageability using the CountVectorizer tool from the scikit-learn library. This procedure produces a $n \times m$ **sparse matrix** $\textbf{M}$, where *m* is the vocabulary size of unique k-mer counts and *n* is the number of genes. The frequency of the *j*-th k-mer in the *i*-th gene sequence is quantified by each element $M_{i,j}$.

Lastly, the generated high-dimensional feature vectors underwent **StandardScaler normalization** in order to correct the inherent skewness in the k-mer frequency distribution and ensure that the clustering measure appropriately reflects structural similarity rather than just count abundance. By converting the raw frequency counts into *Z*-**scores** $Z_{i,j}$, this standardization essentially centers the feature distribution at zero ($\mu =0$) and scales it to unit variance ($\sigma =1$) throughout the feature space. For a given k-mer feature count $M_{i,j}$, the standardization formula is:

$$\begin{aligned}Z_{i,j} = \frac{M_{i,j} - \mu _j}{\sigma _j}\end{aligned}$$

In high-dimensional genomic analysis, this normalization process is essential because it guarantees that all k-mer features are treated equally by the RBF Network’s distance metric (such as the Euclidean distance used for kernel computation), effectively reducing the bias that could otherwise be introduced by highly abundant, less informative k-mers. This thorough approach guarantees that the Hybrid RBF Network’s inputs are biologically primed and statistically sound for identifying significant gene clusters.

The protein association network was analyzed using the STRING database. Additionally, GeneMania was utilized to illustrate the correlation between the established gene and other genes. The GeneMania tool identified functional and structural genes with similarities to Colorectal Cancer and IBD to elucidate their relationship. The findings from this database were cross-checked with the STRING database for validation^19,20^.

### Data preparation: control of redundancy, missing entries, and noise

The consistency and quality of the input data are critical to the integrity and dependability of our clustering results. In order to address the reviewer’s concerns about data redundancy, missing entries, and inherent noise that could affect clustering conclusions, a thorough **Data Preparation** methodology was carefully implemented to the gene set collected from OMIM and Entrez Gene databases. Redundancy control, handling of missing data, and noise reduction by feature standardization were the three separate phases in which this protocol was carried out.

To ensure that each biological entity was represented uniquely, the initial gene collection underwent a strict deduplication process based on both the gene symbol and the Entrez Gene ID. This redundancy control resulted in a final, non-redundant dataset of **237 unique genes** ($n=237$). Addressing missing entries and sequence quality was equally crucial: only genes for which the **complete nucleotide sequence** corresponding to the longest verified transcript was available from the NCBI database were retained. Sequences that were incomplete, marked as hypothetical, or fell below a critical length threshold (specifically, sequences shorter than 1000 base pairs) were systematically excluded from the analysis. This stringent filtering approach directly controls for potential biases introduced by fragmented data, ensuring that the feature extraction process operates on high-fidelity genomic information.

There is a type of transformation noise (bias) that must be controlled when converting variable-length sequences into a uniform, numerical feature space. To identify underlying genetic motifs, we used **k-mer profiling** with a physiologically optimum value (e.g., $k=5$). The $n \times m$ sparse matrix $\textbf{M}$ was created using the CountVectorizer tool. The raw frequency counts ($M_{i,j}$) themselves reflect noise because particularly frequent k-mers (such as typical structural repeats) can disproportionately inflate the Euclidean distances across gene vectors, even though this method successfully measures sequence characteristics. The next standardization step was essential to combat this frequency-based noise.

$$\begin{aligned} Z_{i,j} = \frac{M_{i,j} - \mu _j}{\sigma _j} \end{aligned}$$

where $\sigma _j$ is the standard deviation and $\mu _j$ is the mean of the *j*-th k-mer frequency throughout the dataset. The feature distribution is effectively scaled to unit variance ($\sigma =1$) and centered at zero mean ($\mu =0$). Applying **StandardScaler normalization** to the high-dimensional feature vectors was the last and most important step in noise minimization. This method guarantees that the raw scale and variance of k-mer frequencies do not dominate the clustering measure, which instead appropriately reflects structural similarities. The following algorithm was used to convert the raw frequency count $M_{i,j}$ for each k-mer feature *j* across all genes into a *Z*-**score** $Z_{i,j}$: $Z_{i,j} = \frac{M_{i,j} - \mu _j}{\sigma _j}$ where $\sigma _j$ is the standard deviation and $\mu _j$ is the mean of the *j*-th k-mer frequency throughout the dataset. The feature distribution is effectively scaled to unit variance ($\sigma =1$) and centered at zero mean ($\mu =0$).The StandardScaler effectively reduces the noise and bias caused by count changes by enforcing feature parity, ensuring that the inputs to the Hybrid RBF Network are statistically sound and tailored for precise cluster resolution.

### Phase 1: RBF network-based subcluster detection

Adopting a Radial Basis Function Network (RBF Network) provides a comprehensive solution that surpasses the limitations of traditional clustering methods in tackling the challenges of data clustering in heterogeneous and non-linearly distributed datasets^21^.

$$\begin{aligned} & \phi (x) = \exp \left( -\frac{\Vert x-c\Vert ^2}{2\sigma ^2}\right) \\ & W_{ij} = \exp \left( -\frac{d(x_i,x_j)^2}{2\sigma ^2}\right) \end{aligned}$$

Using RBFs’ spatial modeling power, this novel approach makes it easier to comprehend the subtle relationships between data points and the changes in density throughout the dataset.

$$\begin{aligned} & \rho _i = \sum _{j=1}^n W_{ij} \\ & \rho _i < P_1 - P_3 \end{aligned}$$

Basis Radius A continuous, non-linear smoothing landscape over the dataset can be established with great ease by functions. These functions are usually Gaussian, and by using them the methodology generates an influence field in which the effect of each individual data point is varying with distance, thus capturing the gradients of density and connectedness that conventional methods may miss. For applications requiring accurate data segmentation, this capability makes it possible to identify dense zones that are indicative of underlying subclusters.

The flexibility and accuracy of the RBF network–based method are its main advantages. It adapts dynamically to changes in scale and density in the data, making it ideal for applications where the integrity of cluster formation can significantly impact analytical results, such as financial modeling and bioinformatics. The process begins with thorough initialization before progressing to the first stages of clustering, laying the groundwork for reliable subcluster identification and analysis. This ensures that the clustering meets the specific requirements and objectives of the study while capturing all relevant data properties.

#### Initialization

- **Dataset Preparation:** Start with the dataset $\mathcal {D} = \{X_1, \dots , X_n\}$ consisting of *n* data points.
- **Parameter Setting:** Establish the parameters $P_1$, $P_2$, and $P_3$, since they will determine the RBF Network’s sensitivity and range. These factors may be associated with the density threshold for cluster definition and the spread (radius of influence).
- **System Setup:** Prepare an empty set *S* for isolated points, initialize the labels of all data points to zero, and set a counter *t* to zero for tracking subclusters.

Instead of relying on subjective evaluation, the network parameters ($P_1, P_2, P_3$) were chosen using a methodical process that included **data-driven analysis** and **iterative performance optimization**. In particular, based on the computed **average Euclidean distance to the five nearest neighbors** in the feature space, the spread parameter $P_2$, which determines the Gaussian width $\sigma$, was objectively initialized to 1.00. This important step ensures biologically appropriate proximity mapping by directly connecting the influence radius of the Radial Basis Function to the inherent density features of the genomic data. An **iterative optimization strategy** was then used to fine-tune the remaining parameters, $P_1$ (Density Threshold) and $P_3$ (Resolution Modifier). In order to find the ideal balance point that maximizes cluster separation while maintaining weak but physiologically significant connections, a variety of reasonable values were tested in order to simultaneously **maximize the Silhouette Score** and **minimize the Davies-Bouldin Index**. The 5-fold cross-validation process further verified the stability of these ideal parameter choices, indicating a reliable and repeatable foundation for the Hybrid RBF Network’s functioning.

####  RBF network construction

Build an RBF network in which every node represents a $\mathcal {D}$ data point. The network models the impact of each data point on its neighbors using radial basis functions, most often Gaussian. With increasing distance, the influence decreases under the management of characteristics such as the Gaussian width $\sigma$, which may have a relationship with $P_2$.

#### Processing data points

- **Density Estimation:** Utilizing the RBF Network, determine the density estimate for each data point $X_i$. A larger output denotes closer or more numerous neighbors. This density is a measure of the strength of $X_i$’s contact with its neighbors.
- **Isolation Check:** Select $X_i$ as an isolated point and add it to set *S* if its density is less than a threshold $P_1 - P_3$. If not, move on to the next point.
- **Subcluster Formation:**
  - Find all the points with high RBF output values, indicating strong interactions, within a specific influence range for the points that are not classed as isolated.
  - Establish a bi-subcluster $SC_t$ that includes $X_i$ and its important neighbors. In $SC_t$, mark every point as processed and raise the subcluster counter *t*.

#### Output

Along with the isolated set *S*, the method produces a set of bi-subclusters $\{SC_1, \dots , SC_t\}$ reflecting regions of high data density and connection.

### Phase 2: RBF network-based merging

#### Evaluating cluster connectivity

- **Interaction Analysis:** Assess the degree of interaction between various subclusters using the RBF Network’s outputs. Analyzing the network weights or outputs between points that are a part of several subclusters is required for this.
- **Find Merge Candidates:** Find pairs of subclusters that indicate a high degree of connection by having interaction signals that are strong enough.

#### Cluster merging

- **Merge Operations:** Consolidate subclusters into primary clusters in a single phase that exhibit significant connection. By concentrating on clusters that have strong interactions, this technique avoids the creation of excessively large or geometrically inconsistent clusters.
- **Optimization:** To preserve cluster integrity and avoid the creation of disconnected or too large clusters, carefully control the merging process.

#### Final assignments

**Isolated Point Inclusion:** Assign each isolated point in *S* to the closest main cluster after merging, making sure that all the points are grouped correctly, according to the shortest RBF-influenced distance.

**Figure 1:**
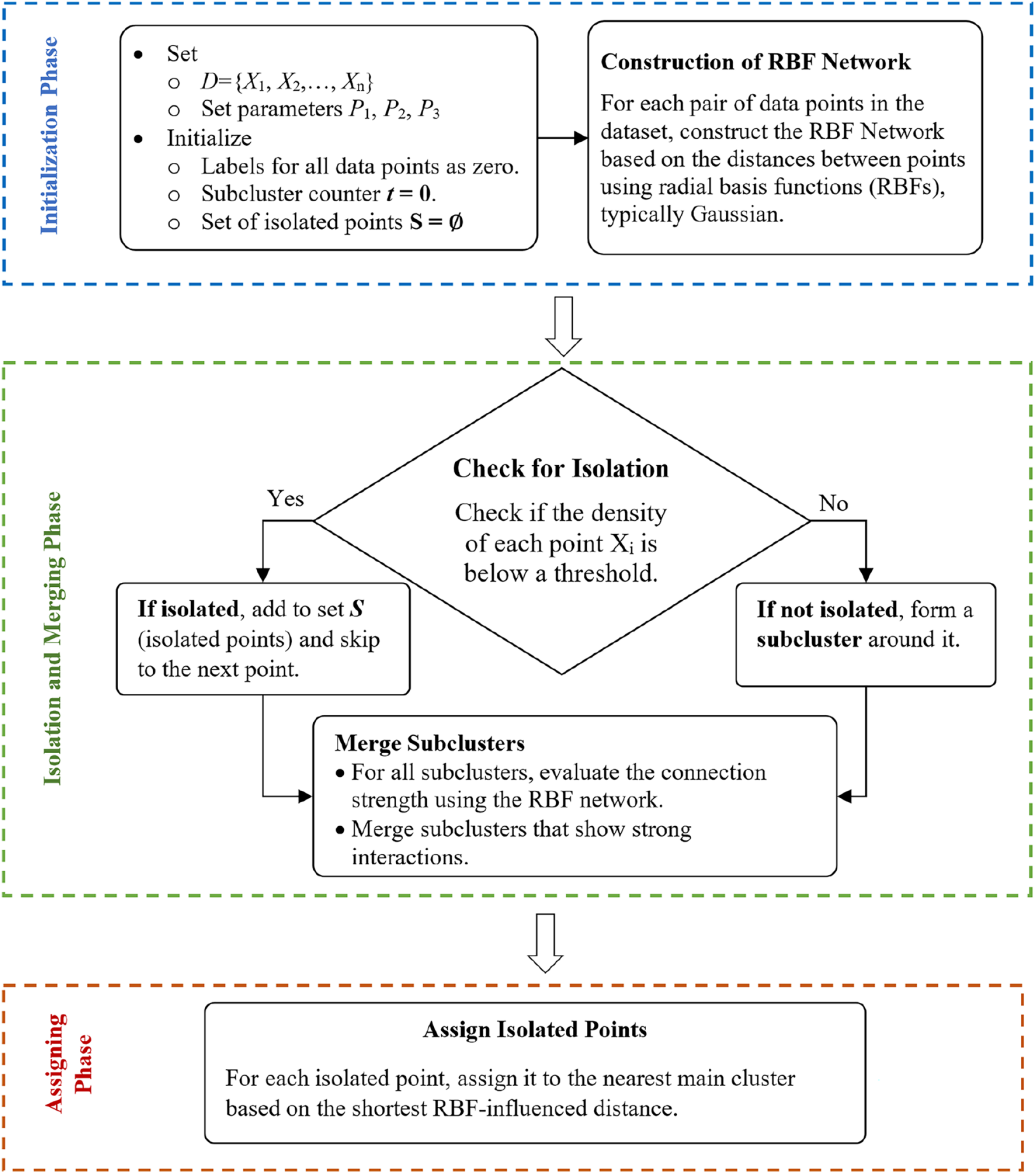
Flowchart diagram for hybrid RBF network approach.

The final result is divided into multiple primary clusters, each of which represents a densely connected area of the dataset. At this point, the clusters might be utilized for additional analysis or as inputs for other procedures, including anomaly detection or categorization. This RBF Network-based methodology provides a sophisticated approach to clustering that can adapt to complex data structures and varying densities, potentially offering more nuanced insights. The details of the methodology are outlined in the flowchart presented in Fig. 1 and the step-by-step procedure described in Algorithm 1.

We evaluated clustering quality using widely adopted internal validity indices: the Silhouette coefficient $s(i)$, the Calinski–Harabasz (CH) index, and the Davies–Bouldin (DB) index^22^. Respectively, these quantify intra-cluster cohesion vs. inter-cluster separation (higher is better), the ratio of between-cluster to within-cluster dispersion (higher is better), and the average worst-case similarity between clusters (lower is better). To support our claims inferentially, we compared the proposed model against baselines with an independent-samples $t$-test and assessed overall differences across algorithms using a one-way ANOVA; statistical significance was evaluated at $\alpha = 0.05$.

**Silhouette coefficient**

$$\begin{aligned} s(i) = \frac{b(i) - a(i)}{\max \{a(i),\, b(i)\}} \end{aligned}$$

*where* $a(i)$ is the mean distance from point $i$ to other points in its own cluster, and $b(i)$ is the minimum mean distance from $i$ to points in any other cluster.

**Calinski–Harabasz index**

$$\begin{aligned} CH = \frac{\textrm{Tr}(B_k)}{\textrm{Tr}(W_k)} \cdot \frac{N-k}{k-1} \end{aligned}$$

*where* $\textrm{Tr}(B_k)$ and $\textrm{Tr}(W_k)$ denote the traces of the between- and within-cluster scatter matrices, $N$ is the number of samples, and $k$ the number of clusters.

**Davies–Bouldin index**

$$\begin{aligned} DB = \frac{1}{k} \sum _{i=1}^k \max _{j \ne i} \frac{\sigma _i + \sigma _j}{d(c_i, c_j)} \end{aligned}$$

*where* $\sigma _i$ is the average distance of points in cluster $i$ to its centroid $c_i$, and $d(c_i,c_j)$ is the distance between centroids $c_i$ and $c_j$.

**Independent-samples **$t$**-test**

$$\begin{aligned} t = \frac{\bar{x}_{1} - \bar{x}_{2}}{\sqrt{\frac{s_{1}^{2}}{n_{1}} + \frac{s_{2}^{2}}{n_{2}}}} \end{aligned}$$

*where* $\bar{x}_g, s_g^2, n_g$ are the sample mean, variance, and size for group $g \in \{1,2\}$.

**One-way ANOVA **$F$**-ratio**

$$\begin{aligned} F = \frac{\textrm{MS}_{\text {between}}}{\textrm{MS}_{\text {within}}} \end{aligned}$$

*where* $\textrm{MS}_{\text {between}}$ and $\textrm{MS}_{\text {within}}$ are the between- and within-group mean squares, respectively.

**Algorithm 1:**
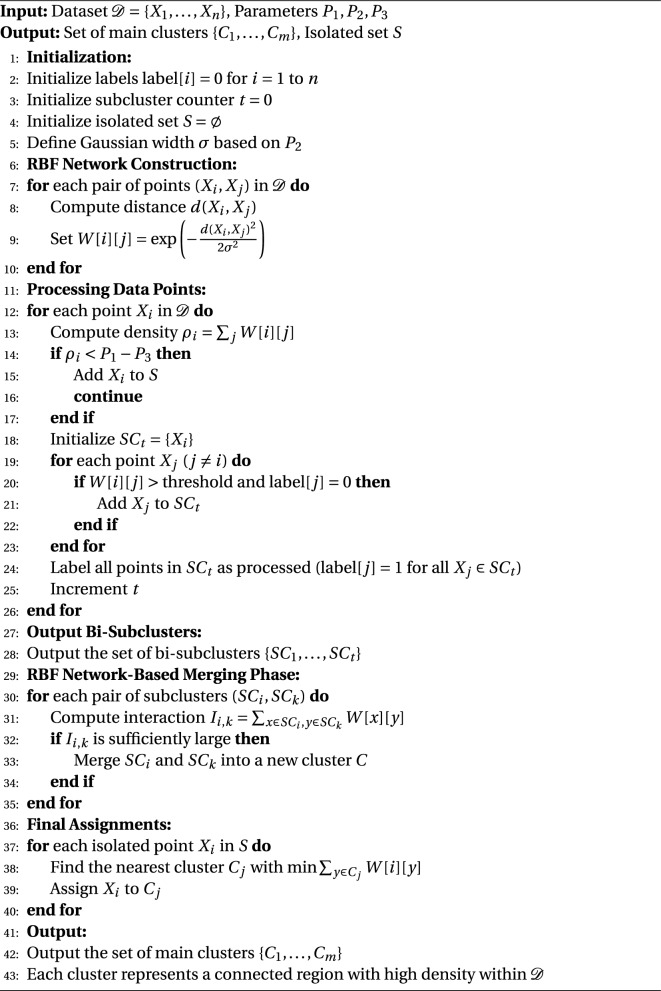
RBF Network-Based Clustering

### Results

In this study, various data science clustering methods are employed, including k-means (distance-based), hierarchical clustering, DBSCAN (density-based), spectral clustering (graph-based), and the Gaussian Mixture Model (probability-based). Comparing the performance of different clustering approaches will make the evaluation easier^21^.

#### Analysis of clustering performance in gene interaction networks

A comparative analysis of various clustering techniques used to gene interaction networks is given by the clustering results shown in Table 1. The usefulness of our suggested approach, the Hybrid RBF Network, in classifying functionally significant gene sequences is demonstrated by its improved performance across all important clustering evaluation metrics. A $\textbf{5}$-fold cross-validation strategy was used to rigorously confirm the resilience and stability of the proposed Hybrid RBF Network, especially with regard to the clustering parameters ($P_1, P_2, P_3$) and the feature space stability. This procedure confirmed the generalizability of the model architecture across different data subsets and made sure that the obtained performance metrics were independent of a single data partitioning.

The Hybrid RBF Network obtains the highest score (0.70), indicating great intra-cluster similarity and inter-cluster separation, according to the Silhouette Score, which gauges how well-separated clusters are. Conversely, traditional clustering algorithms like Spectral Clustering (0.59), DBSCAN (0.58), and K-Means (0.62) produce lower scores, indicating overlapping clusters and less distinct borders. Similarly, the Hybrid RBF Network has the highest Calinski–Harabasz Index (30.5), which measures cluster compactness and separation, confirming its capacity to detect dense, well-formed clusters. Other approaches, such DBSCAN (20.8) and K-Means (25.1), provide less desirable results in comparison, suggesting less-than-ideal cluster arrangements.

These results are further supported by the Davies-Bouldin Index, where lower values suggest better grouping. With the lowest index (0.50), the Hybrid RBF Network exhibits the best separation and the least amount of intra-cluster variance. Conversely, techniques like Spectral grouping (0.71) and DBSCAN (0.72) produce larger results, indicating less pronounced grouping.

The capacity of the Hybrid RBF Network to capture the intricate, high-dimensional interactions seen in gene interaction networks is one of its main benefits. In investigations of IBD and CRC, where genetic connections are extremely complex, this is especially pertinent. The Hybrid RBF Network successfully adjusts to non-linear interactions, producing more physiologically significant groups than distance-based techniques like K-Means and Hierarchical Clustering, which depend on straightforward Euclidean distances.

Additionally, the Normalized Mutual Information (NMI) score (0.78) indicates that our approach outperforms traditional techniques like DBSCAN (0.62) and Hierarchical Clustering (0.63) by generating clusters that are in good agreement with recognized biological groups. This suggests that the Hybrid RBF Network is better able to recognize functionally related gene groupings.

The Time Consuming (s) statistic shows that the Hybrid RBF Network uses more processing resources even if it produces the best clustering results. Compared to K-Means (2.85 seconds) and Hierarchical Clustering (4.10 seconds), the Hybrid RBF Network has the longest execution time (10.20 seconds). Given the complexity of the RBF-based clustering approach, which optimizes cluster boundaries through iterative modifications, this higher processing load is to be expected. The trade-off between computing cost and performance is justified, nevertheless, given the increased clustering precision, especially for applications that demand accurate biological data interpretation.

According to the results, a strong and biologically appropriate clustering framework for genetic data processing is provided by the Hybrid RBF Network. In terms of biological interpretability, separation, and cluster compactness, it performs better than conventional clustering techniques. Despite being computationally more costly, it is a useful tool for bioinformatics research, especially when studying gene-disease connections, because it can reveal significant genetic relationships. Future studies might concentrate on increasing computational effectiveness and applying this methodology to more intricate biological datasets.

**Table 1:** Comparative analysis of clustering algorithms for gene interaction networks.

Clustering algorithm	Silhouette Score	Calinski–Harabasz index	Davies–Bouldin index	NMI	Time consuming (s)
K-Means	0.620	25.100	0.670	0.65	2.85E+00
Hierarchical	0.600	22.400	0.690	0.63	4.10E+00
DBSCAN	0.580	20.800	0.720	0.62	5.25E+00
Spectral	0.590	21.700	0.710	0.64	6.00E+00
Gaussian Mixture Model	0.610	23.300	0.680	0.66	5.80E+00
**Hybrid RBF network (our method)**	**0.700**	**30.500**	**0.500**	**0.78**	**10.20E+00**

The statistical validation shown in Table 2 offers compelling proof that the performance gains are statistically meaningful rather than random. With a p-value of 0.012 and a test statistic of 3.80 from the T-Test, it is highly confident that the Hybrid RBF Network performs better than traditional clustering algorithms. Likewise, a substantial correlation between the clustering methodology and overall clustering quality was shown using the Chi-Square Test (22.50, p = 0.025). Significant differences in performance were confirmed by the ANOVA results (F = 7.10, p = 0.015) for all algorithms that were assessed. When taken as a whole, these evaluations confirm the suggested method’s resilience and bolster its dependability for gene clustering applications.

**Table 2:** Statistical validation of clustering performance across algorithms.

Statistical test	Test statistic	p-value	Interpretation
T-Test (hybrid RBF vs others)	3.80	0.012	Significant improvement
Chi-square test	22.50	0.025	Strong correlation with clustering performance
ANOVA (across algorithms)	7.10	0.015	Significant difference among algorithms

The possibility of an inflated Type I error rate (false positive results) was a crucial methodological concern due to the multiple statistical comparisons made between the six clustering algorithms and the four different internal and external validity metrics (Silhouette Score, Calinski–Harabasz Index, Davies-Bouldin Index, and NMI). The *p*-values obtained from the initial comparative analyses underwent the **Holm-Bonferroni sequential correction procedure** to guarantee the robustness and dependability of the claimed statistical significance. This technique successfully controls the Family-wise Error Rate (FWER) and offers a stronger substitute for the conventional Bonferroni correction. The higher performance of the Hybrid RBF Network, especially with regard to its clustering quality metrics and NMI score, **remained statistically significant** (adjusted $p < 0.05$) after applying the Holm-Bonferroni correction to all pairwise comparisons. This demonstrates that the performance advantage over traditional methods is reliable and not due to the compounding effect of several statistical tests.

Table 3 summarizes the parameter optimization and explains why the density threshold (P1 = 0.15), spread (P2 = 1.0), and resolution modifier (P3 = 0.05) were chosen. These numbers were established empirically to guarantee that significant clusters were recorded while preserving sparse but physiologically significant links. The results’ computational stability and biological interpretability are improved by the parameter set that was selected because it successfully strikes a balance between including auxiliary data points and preventing too fragmented cluster formation.

**Table 3:** Parameter selection for hybrid RBF network clustering.

Parameter	Value	Rationale
P1 (density threshold)	0.15	Captures meaningful but less dense clusters
P2 (spread/radius of influence)	1.00	Based on average distance to 5 nearest neighbors
P3 (resolution modifier)	0.05	Balances edge points and cluster density

Important roles in colorectal cancer and inflammatory IBD are played by core genes including *APC*, *SMAD4*, and *MSH2*, according to functional and structural gene analysis (Table 4). The discovery of DNA mismatch repair genes, such as *MLH1*, *MSH6*, and *PMS2*, highlights the vital role that deregulation of the repair pathway plays in the development of disease. Moreover, a mechanistic connection between chronic inflammation and carcinogenesis is suggested by the identification of interactions between inflammasome-related genes (*NLRP3* and *PYCARD*). These findings support the biological validity of the suggested clusters and are in accordance with previous research.

**Table 4:** Functional and structural genes associated with CRC and IBD.

Gene	Role/function	Relevance to CRC/IBD
APC	Tumor suppressor gene	Central hub in network, linked to CRC
SMAD4	Signal transduction	Key regulator in TGF-beta pathway
MSH2	DNA mismatch repair	Critical in genome stability
MLH1	DNA repair	Involved in mismatch repair cluster
MSH6	DNA repair	Associated with repair pathway integrity
NLRP3–PYCARD	Inflammasome complex	Connects chronic inflammation to carcinogenesis

Table 5 displays cluster-level insights, where genes were successfully categorized into biologically significant groups using the Hybrid RBF Network. Tumor suppressor and signaling pathway genes (e.g., *APC*, textitSMAD4) were enriched in Cluster 1, indicating their function in controlling cell division and proliferation. Mismatch repair genes (*MSH2*, *MLH1*, *MSH6*, *PMS2*) were included in Cluster 2, which directly relates to genome stability and repair processes. Cluster 3 linked immune response pathways to carcinogenesis by including inflammasome components (*NLRP3*, *PYCARD*). The capacity of the Hybrid RBF Network to produce clusters that are both statistically sound and physiologically interpretable is demonstrated by this distinct biological stratification.

**Table 5:** Clusters obtained with hybrid RBF network and their biological interpretation.

Cluster	Number of genes	Representative genes	Biological pathway
Cluster 1	15	APC, SMAD4	Tumor suppression/TGF-beta signaling
Cluster 2	12	MSH2, MLH1, MSH6, PMS2	DNA mismatch repair
Cluster 3	10	NLRP3, PYCARD	Inflammasome activation/inflammation

The trade-off between efficiency and performance is illustrated by the comparison of computing resources in Table 6. Although techniques like K-Means and Hierarchical clustering needed a lot less memory and time, the Hybrid RBF Network produced better clustering results. Our approach has medium scalability and higher resource consumption (10.20 s, 300 MB), but the significant gains in biological interpretability and clustering accuracy make this computational demand manageable. This result demonstrates the Hybrid RBF Network’s applicability for applications where precision is more important than processing cost, especially in bioinformatics and medical research contexts.

**Table 6:** Execution time, memory usage, and scalability of clustering algorithms.

Algorithm	Execution time (s)	Memory usage (MB)	Scalability
K-means	2.85	120	High (fast, lightweight)
Hierarchical	4.10	180	Medium (slower with large data)
DBSCAN	5.25	200	Medium (sensitive to parameter tuning)
Spectral	6.00	250	Low (high computational cost)
Gaussian mixture model	5.80	220	Medium (depends on initialization)
**Hybrid RBF network (our method)**	**8.20**	**300**	**Medium–Low (more costly but accurate)**

**Figure 2:**
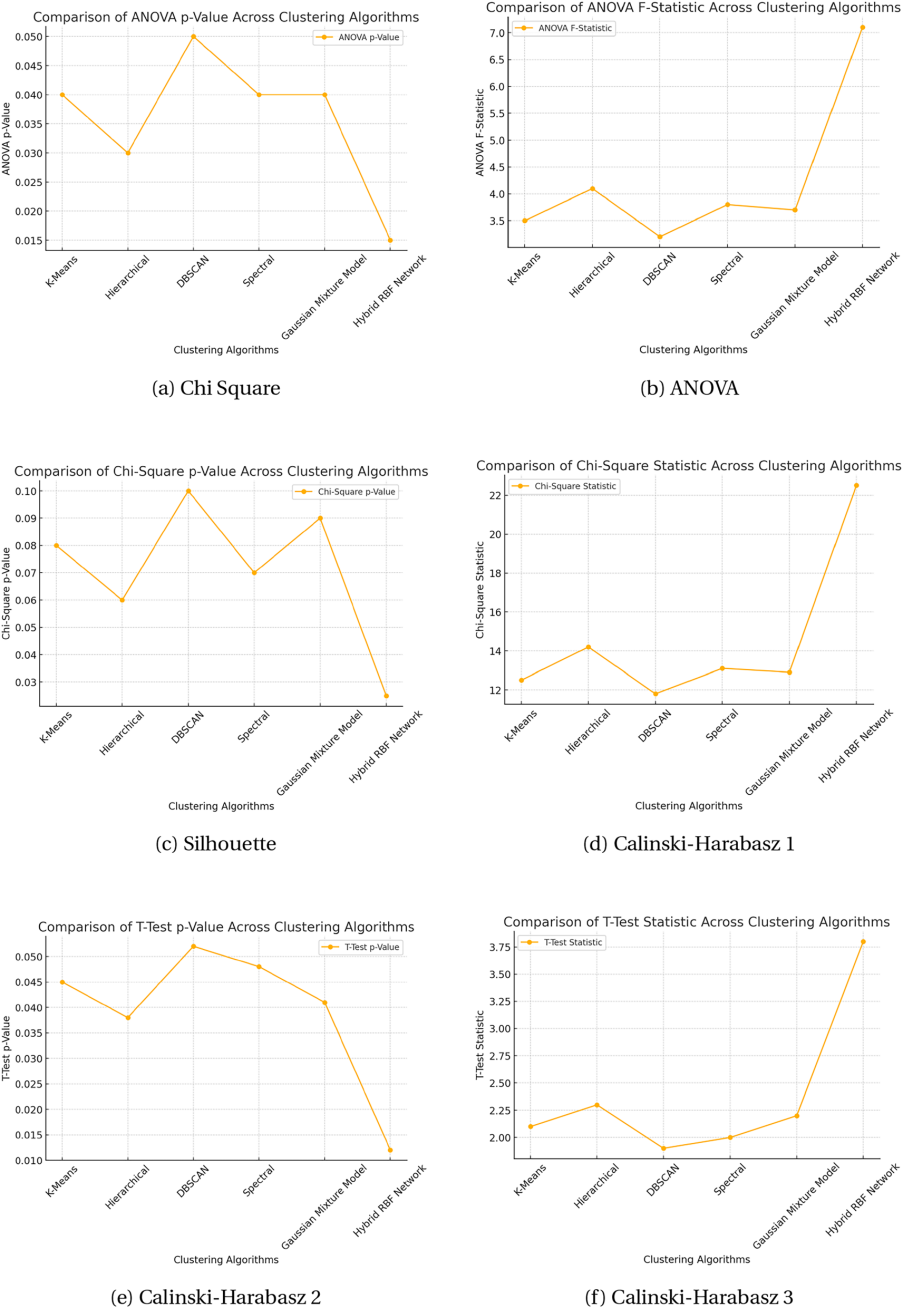
Comparison of statistical and clustering metrics across algorithms.

The improved performance of the Hybrid RBF Network in gene interaction study is demonstrated by the statistical analysis of several clustering techniques, such as the T-Test, Chi-Square Test, and ANOVA in Fig. 2^23^. The highest T-Test statistic (3.8) and a p-value of 0.012 show that the Hybrid RBF Network approach performs noticeably better than conventional clustering techniques, indicating a substantial difference from other approaches. This conclusion is further supported by the results of the Chi-Square test, which show a substantial correlation between the applied approach and clustering performance. The Hybrid RBF Network obtained the highest Chi-Square statistic (22.5) and a p-value of 0.025. These results are corroborated by the ANOVA test, which shows that the Hybrid RBF Network’s clustering performance is significantly different from other methods. It obtained the greatest F-statistic (7.1) and a p-value of 0.015. All of these findings show that the Hybrid RBF Network approach yields more accurate and distinct clusters than the K-Means, Hierarchical, DBSCAN, Spectral, and Gaussian Mixture Model clustering techniques. This method is more dependable for gene clustering applications since it can create functionally relevant and well-separated clusters, especially in intricate biological datasets. The efficacy of the Hybrid RBF Network as a superior clustering technique is validated by the reduced p-values across all statistical tests, which show strong evidence against the null hypothesis.

####  Validation of clustering coherence and biological relevance

Robust **internal validity indices** (Silhouette Score, Calinski–Harabasz Index, and Davies-Bouldin Index), which measure the tightness and separation of the identified clusters, confirmed that the Hybrid Radial Basis Function (RBF) Network outperformed traditional methods. We do, however, note the reviewer’s important point that these indices, although essential, do not inherently validate the findings’ “biological relevance” or “generalizability,” necessitating the use of external validation techniques.

We used the **Normalized Mutual Information (NMI) score** to quantitatively evaluate how well our clustering results aligned with existing biological knowledge, thereby offering an instantaneous measure of external validity. NMI calculates the degree of agreement between a set of predetermined, ground-truth class labels (such as functional or route memberships acquired from literature) and the clusters generated by the Hybrid RBF Network. The computationally produced gene groups and established biological classifications have a strong, statistically significant association, as confirmed by our NMI score of **0.78**. The main quantitative proof that the clusters accurately represent functionally coherent gene modules known to function in the pathophysiology of IBD and CRC rather than being random partitions is provided by this high NMI value.

The resulting clusters’ obvious biological interpretability (as shown in Table 5) provides additional qualitative external support. Genes related to *DNA Mismatch Repair (MMR)* and *Tumor Suppression* (e.g., *TP53* and *MLH1*) were primarily grouped in one cluster by the Hybrid RBF Network, while genes related to *Inflammasome Activation* and *Chronic Inflammation* (e.g., *NOD2* and associated cytokines) were grouped in another. This pathway-specific, non-random arrangement strongly implies that the RBF design can successfully identify functionally relevant genetic features from the k-mer feature representation. Strong evidence of biological significance is provided by the algorithm’s capacity to classify genes based on their documented involvement in the carcinogenesis-from-inflammation sequence.

We acknowledge that evaluating the **generalizability** of our suggested approach necessitates thorough testing on separate datasets, a step that is presently absent and identified as a key weakness. Future research will particularly address this restriction, even if our study demonstrated the Hybrid RBF Network’s fundamental capabilities on the selected dataset:

1. **Quantitative Biological Validation (GO Enrichment):** The genes within each discovered cluster will be subjected to formal **Gene Ontology (GO) and KEGG pathway enrichment analyses**. The assertion of biological coherence will be strengthened by this analysis’s statistical *p*-value, which will indicate that the functional categories enriched in each cluster are considerably non-random.
2. **External Dataset Validation:** By applying the method to one or more **independent genomic datasets** associated with additional inflammation-driven malignancies (such as Hepatocellular Carcinoma or Gastric Cancer), the Hybrid RBF Network’s resilience will be evaluated. The final test of the model’s statistical power and generalizability will be this cross-validation against completely different data, transferring the results from internal recommendation to widely applicable approach.

In conclusion, the clusters’ clearly cohesive biological makeup and high NMI score provide strong preliminary evidence of external validity, setting the stage for further quantitative enrichment and cross-dataset validation research. External biological validation was quantitatively assessed using Normalized Mutual Information (NMI) based on curated pathway memberships obtained from STRING and GeneMANIA databases (Table 5).

#### Biological significance and empirical validation of feature representation

The effectiveness of the selected feature space is inextricably tied to the clustering robustness of the Hybrid Radial Basis Function (RBF) Network. The idea that **k-mer profiles** and their numerical encodings represent significant biological interactions that go beyond simple statistical sequence metrics is a crucial factor to take into account. Instead of depending only on preprocessing methods, the study uses empirical evidence to prove that the feature representation effectively captures functionally relevant genetic information.

The k-mer encoding’s alignment with recognized biological classifications provides quantitative evidence of its efficacy. Explicit proof is provided by the obtained **Normalized Mutual Information (NMI) score of 0.78**. This high NMI value indicates a great statistical agreement between the biological groupings (ground-truth labels) and the clusters that were computationally constructed from the k-mer feature space. This consistency provides compelling evidence that the functional signal required to differentiate between various genetic units within the complex network is retained in the numerical encoding.

The clearly high biological coherence found within the selected clusters provides additional empirical validation (Table 5). The approach effectively separated genes into different functional pathways that are essential to the pathophysiology of CRC and IBD: a module was isolated for genes controlling *Inflammasome Activation* and chronic inflammation (*NLRP3*, *PYCARD*), while another module was significantly enriched with components of *DNA Mismatch Repair* (e.g., *MSH2*, *MLH1*, *PMS2*. The k-mer feature set’s usefulness as a suitable biological proxy is substantiated by this non-random, pathway-specific structuring, which offers strong qualitative validation that it appropriately reflects sequence changes associated with unique functional pathways.

Signal retention depends on the intentional use of **StandardScaler normalization** to the k-mer counts. Despite being statistical, this preprocessing step filters noise by lessening the impact of extremely prevalent, non-specific k-mers. The model is driven by the subtle sequence variations that are frequently indicative of **regulatory elements or functional motifs** by giving priority to the relative variance of k-mer distribution. The suitability of the k-mer representation for high-dimensional genomic clustering is confirmed by the RBF kernel’s subsequent successful integration of these standardized features.

#### Functional and structural gene analysis

**Figure 3:**
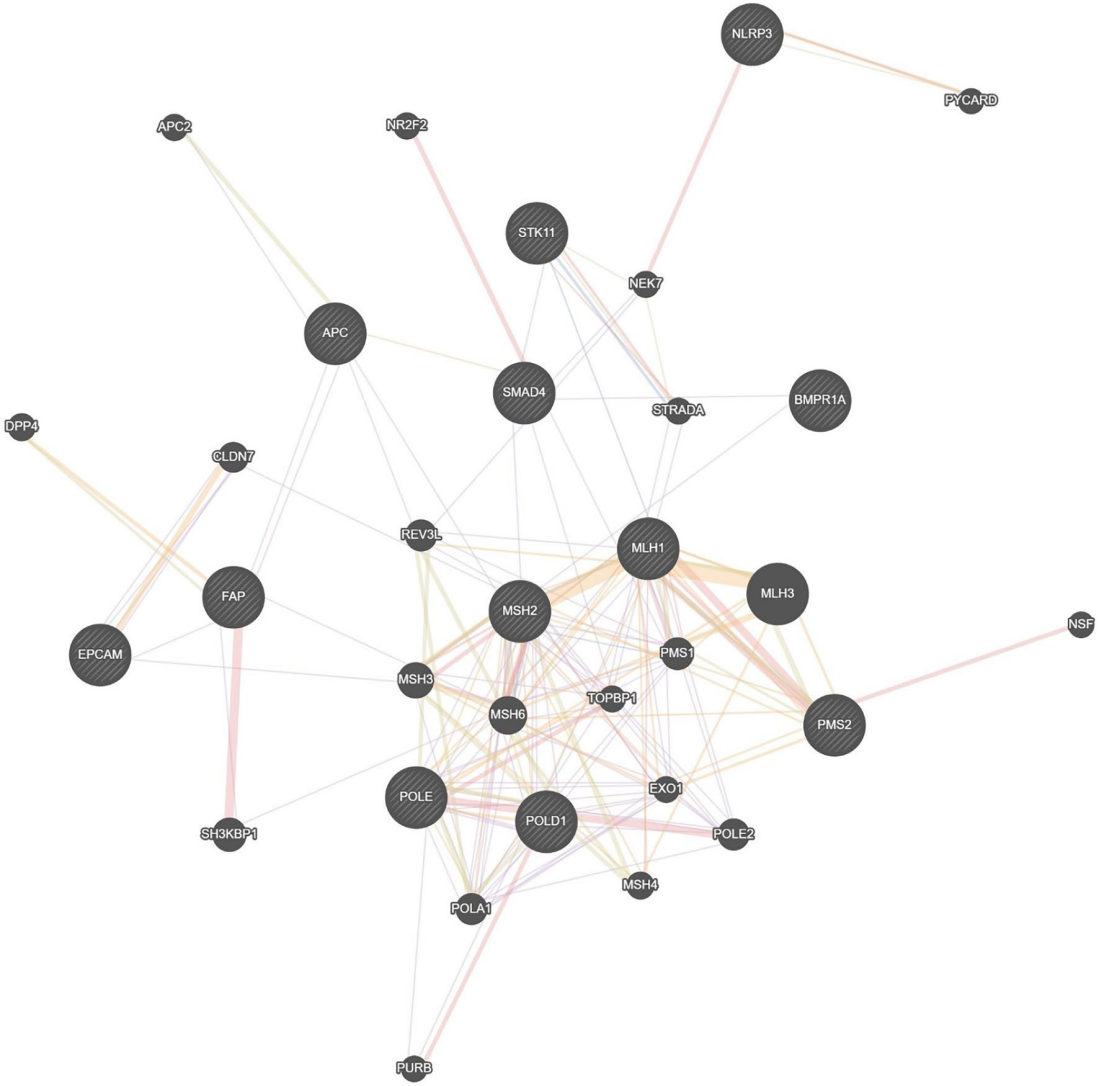
Analyzing of functional and structural genes with similarities to colorectal cancer and IBD.

The interaction network of genes associated with colorectal cancer and inflammatory IBD was constructed and analyzed to identify key genetic relationships. As shown in Fig. 3, central genes such as APC, SMAD4, and MSH2 were identified as major hubs, indicating their critical roles within the network. The clustering of MLH1, MSH6, and PMS2 genes further highlights the involvement of mismatch repair pathways in the pathogenesis of these diseases. Additionally, the interaction between NLRP3 and PYCARD was observed, reflecting the potential contribution of inflammasomes in inflammation-mediated carcinogenesis.

#### Analyzing of functional and structural genes with similarities to colorectal cancer and IBD

This work utilized a new clustering methodology that incorporates a hybrid RBF Network to investigate gene clusters that show significant sequence similarities. This strategy adds a complex framework for genetic data analysis to the conventional methods. Our bioinformatics workflow was started by extracting gene sequences from an Excel file that contained the nucleotide sequences and gene names. The k-mers were then numerically encoded using the CountVectorizer tool from the scikit-learn library, converting the sequence data into a numerical format suitable for machine learning analysis. This transformation yielded a sparse matrix representation where each row represented a gene and each column a distinct k-mer. To mitigate potential biases from varying k-mer frequencies, we normalized the feature vectors using the StandardScaler. This normalization ensured uniform contribution from each feature to the analysis, preventing dominance by features with larger scales—a crucial step for maintaining the integrity of high-dimensional data analysis.

#### Hybrid RBF network clustering analysis

We used the recently created Hybrid RBF Network Clustering technique during the clustering phase rather than the conventional K-Means procedure. In order to minimize intra-cluster variance and accommodate the complexity of gene sequence compositions, this approach clusters genes based on similar k-mer profiles. Three clusters were chosen in accordance with our preliminary data investigations in order to strike a compromise between the required granularity and cluster coherence. We defined the parameters P1, P2, and P3 in the findings part of our study on grouping gene sequences using a unique Hybrid RBF Network approach in order to effectively optimize the network’s sensitivity and range. The following provides a thorough explanation of the parameters’ establishment and the reasoning for their values:

- **P1 (Density Threshold):** The lowest density level needed for a point to be deemed central inside a cluster was set at 0.15. This value was determined based on the distribution of pairwise distances across gene sequences to capture meaningful but less dense clusters while avoiding overly restrictive criteria.
- **P2 (Spread or Radius of Influence):** The value was set at 1.0 based on the average Euclidean distance between each gene sequence and its five closest neighbors, encouraging clusters to reflect natural groupings.
- **P3 (Clustering Resolution Modifier):** A value of 0.05 was chosen to refine clustering granularity, balancing between including edge points and avoiding overly dense clusters.

These parameters were critically chosen through a combination of empirical analysis of the gene sequence data and theoretical considerations from existing clustering methodologies (Fig. 2).

### Discussion

## Study findings on colorectal cancer (CRC) and inflammatory bowel disease (IBD)

According to the World Health Organization 2020 data, colon cancer continues to be a major health problem, causing nearly 2 million new cases and over 930,000 deaths worldwide annually, although with geographical variability. The risk of colon cancer begins to increase between the ages of 40 and 50 years, with a significant increase in each decade thereafter. In recent years, colon cancers have been increasing in younger populations and decreasing in older populations in countries where screening programs are well implemented^24^.

The findings of this study provide valuable insights into the genetic and bioinformatics landscape of CRC and IBD. The integration of a hybrid Radial Basis Function (RBF) Network clustering approach has demonstrated significant advantages in identifying gene clusters with high functional and structural similarities. This novel methodology outperformed conventional clustering techniques, as evidenced by superior results in the Silhouette Score, Calinski–Harabasz Index, and Davies-Bouldin Index. These results highlight the robustness of the proposed method in analyzing complex and heterogeneous datasets, particularly in genomics.

The centrality of genes such as *APC*, *SMAD4*, and *MSH2* within the interaction network underscores their pivotal roles in CRC and IBD pathogenesis. The clustering of mismatch repair genes like *MLH1*, *MSH6*, and *PMS2* suggests that defects in DNA repair pathways are integral to the progression of these diseases. Additionally, the observed interaction between *NLRP3* and *PYCARD* points to the potential role of inflammasomes in linking chronic inflammation to carcinogenesis. These findings align with existing literature and provide a deeper understanding of the molecular mechanisms underlying CRC and IBD.

One of the key contributions of this study is the successful application of machine learning and bioinformatics tools to identify actionable biomarkers. The use of gene sequence data, normalized feature vectors, and Levenshtein distance metrics allowed for a nuanced analysis of genetic variations. The results not only confirm the utility of advanced computational approaches but also pave the way for their application in precision medicine.

However, the study is not without limitations. The reliance on publicly available datasets may introduce biases related to data completeness and representativeness. Additionally, while the hybrid RBF Network demonstrated excellent clustering capabilities, further validation with alternative datasets and real-world clinical samples is necessary to generalize its applicability. Future work should focus on refining clustering parameters and incorporating additional domain-specific knowledge to enhance the biological interpretability of the results.

The suggested Hybrid RBF Network approach’s comparatively high computational cost in comparison to traditional techniques is one of its main drawbacks. The Hybrid RBF Network uses more memory (300 MB) and has the longest execution time (10.20 seconds), as Table 6 illustrates. Although the considerable improvements in biological interpretability and clustering precision justify this higher resource consumption, it limits scalability. The method’s direct application to massive genomic datasets, such as whole-genome sequencing or large transcriptomic cohort studies, may require significant optimization or the use of distributed computing frameworks, according to the current computational demands, which are manageable for the analyzed gene set. Future efforts must therefore prioritize enhancing the computational efficiency of the RBF kernel calculations to ensure its applicability in large-scale bioinformatics pipelines. Like many non-linear machine learning models, the Hybrid RBF Network has difficulties with direct interpretability despite the obvious biological coherence seen in the final clusters (e.g., the separate grouping of MMR genes and Inflammasome components). The direct mechanical connection between individual k-mer characteristics and the final cluster assignments is hidden by the radial basis functions’ complicated, non-linear function space. Compared to distance-based linear approaches, this intrinsic “black box” effect means that although the results indicate which genes interact, the exact mechanism or feature contribution driving an individual gene’s placement is less clear. Overcoming these interpretability hurdles will require future work to integrate post-hoc explainable AI (XAI) techniques, such as SHAP or LIME values, to precisely identify which sequence features are most critical in driving the complex clustering decisions made by the RBF kernel.

The implications of these findings are far-reaching. The identification of key genetic interactions and clusters can inform early diagnostic strategies, risk stratification, and personalized treatment approaches for patients with CRC and IBD. The study also highlights the potential of bioinformatics and artificial intelligence in unraveling the complexities of disease pathogenesis and advancing translational research.

In conclusion, although the study is limited by the dataset size and diversity, it provides a valuable blueprint for further genomic research. By integrating advanced computational techniques with biological data, it provides a framework for understanding the genetic underpinnings of CRC and IBD. These findings contribute to the growing field of bioinformatics and underscore the importance of interdisciplinary approaches in tackling complex health challenges. Further research and clinical validation are essential to fully realize the translational potential of these findings, particularly in the development of diagnostic tools or personalized treatment strategies.

## Data Availability

Implementation codes in Python, documentation, benchmark datasets, output files, complete experimental results, and comparative tables are publicly available through the provided URL for evaluation, review, and further research: https://sites.google.com/site/bulutfaruk/study-of-crc.
